# Inequality of household water security follows a Development Kuznets Curve

**DOI:** 10.1038/s41467-022-31867-3

**Published:** 2022-08-08

**Authors:** Feng Mao, Joshua D. Miller, Sera L. Young, Stefan Krause, David M. Hannah, HWISE Research Coordination Network, Alexandra Brewis, Alex Trowell, Amber L. Pearson, Amber Wutich, Andrea Sullivan, Asher Y. Rosinger, Ashley Hagaman, Cassandra Workman, Chad Staddon, Desire Tshala-Katumbay, Divya Krishnakumar, Ellis Adams, Ernesto C. Sánchez-Rodriguez, Genny Carrillo, Gershim Asiki, Hala Ghattas, Hassan Eini-Zinab, Hugo Melgar-Quiñonez, Farooq Ahmed, Javier Moran-Martinez, Jonathan Maupin, Jorge Escobar-Vargas, Justin Stoler, Jyoti Mathad, Kelly Chapman, Kenneth Maes, Luisa Samayoa-Figueroa, Mahdieh Sheikhi, Mallika Alexander, Marianne V. Santoso, Matthew C. Freeman, Michael J. Boivin, Milton Marin Morales, Mobolanle Balogun, Monet Ghorbani, Nasrin Omidvar, Nathaly Triviño, Nicola Hawley, Patrick Mbullo Owuor, Raymond Tutu, Roseanne C. Schuster, Sabrina Rasheed, Shalean M. Collins, Sonali Srivastava, Stroma Cole, Wendy Jepson, Yihenew Tesfaye, Zeina Jamaluddine

**Affiliations:** 1 School of Earth and Environmental Sciences, Cardiff University, Cardiff, UK; 2 University of North Carolina at Chapel Hill, Chapel Hill, NC USA; 3 Department of Anthropology, Northwestern University, Evanston, IL USA; 4 Institute for Policy Research, Northwestern University, Evanston, IL USA; 5 School of Geography, Earth and Environmental Sciences, and Birmingham Institute of Forest Research, University of Birmingham, Birmingham, UK; 6 LEHNA - Laboratoire d’ecologie des hydrosystemes naturels et anthropises, University of Lyon, Lyon, France; 7 Arizona State University, Tempe, AZ USA; 8 University of Amsterdam, Amsterdam, The Netherlands; 9 Michigan State University, East Lansing, MI USA; 10 University of Miami, Coral Gables, FL USA; 11 Pennsylvania State University, University Park, Pennsylvania, PA USA; 12 Yale University, New Haven, CT USA; 13 University of North Carolina at Greensboro, Greensboro, NC USA; 14 University of the West of England, Bristol, UK; 15 Oregon Health and Science University, Portland, OR USA; 16 Anode Governance Lab, Bengaluru, India; 17 University of Notre Dame, Notre Dame, IN USA; 18 Hospital Agustin O’Horan, Mérida, Yucatan, Mexico & Universidad Nacional Autonoma de, Mexico, Mexico; 19 Texas A&M University, College Station, TX USA; 20 African Population and Health Research Center, Nairobi, Kenya; 21 American University of Beirut, Beirut, Lebanon; 22 Shahid Beheshti University of Medical Sciences, Tehran, Iran; 23 McGill University, Ste-Anne-de-Bellevue, Quebec, Canada; 24 Quaid-I-Azam University Islamabad, Islamabad, Pakistan; 25 Autonomous University of Coahuila, Coahuila, Mexico; 26 Pontificia Universidad Javeriana, Bogotá, Colombia; 27 Weill Cornell Medicine, New York, NY USA; 28 University of Florida, Gainesville, FL USA; 29 Oregon State University, Corvallis, OR USA; 30 Johns Hopkins University-Byramjee Jeejeebhoy Medical College Clinical Trials Unit, Pune, India; 31 Emory University, Atlanta, GA USA; 32 Universidad Autónoma del Beni José Ballivián, Trinidad, Bolivia; 33 College of Medicine of the University of Lagos, Lagos, Nigeria; 34 Delaware State University, Dover, DE USA; 35 International Center for Diarrhoeal Disease Research Bangladesh, Mohakhali, Dhaka Bangladesh; 36 Tulane University, New Orleans, LA USA; 37 University of Westminster, London, UK; 38 Bahir Dar University, Bahir Dar, Ethiopia; 39 London School of Hygiene & Tropical Medicine, London, UK

**Keywords:** Hydrology, Water resources, Geography

## Abstract

Water security requires not only sufficient availability of and access to safe and acceptable quality for domestic uses, but also fair distribution within and across populations. However, a key research gap remains in understanding water security inequality and its dynamics, which in turn creates an impediment to tracking progress towards sustainable development. Therefore, we analyse the inequality of water security using data from 7603 households across 28 sites in 22 low- and middle-income countries, measured using the Household Water Insecurity Experiences Scale. Here we show an inverted-U shaped relationship between site water security and inequality of household water security. This Kuznets-like curve suggests a process that as water security grows, the inequality of water security first increases then decreases. This research extends the Kuznets curve applications and introduces the Development Kuznets Curve concept. Its practical implications support building water security and achieving more fair, inclusive, and sustainable development.

## Abstract (web summary)

A new study considering data from 7603 households across 28 sites in 22 low- and middle-income countries show that inequality of household water security follows a Development Kuznets Curve.

## Introduction

Water security is a major challenge to achieving the Sustainable Development Goals^1,2^. Billions of individuals worldwide currently live in areas with insufficient water availability, poor water access, unsatisfactory water quality, and/or excessive water-related risks^3–6^. Moreover, climate change, altered water demands through population growth and displacement, and poor water governance also threaten to further exacerbate entrenched water inequalities^7^. As such, the 2019 United Nations World Water Development Report addresses the urgent necessity to consider the demand of disadvantaged groups when managing water resources^8^. Likewise, the 2019 Human Development Report encourages researchers and policymakers to consider human development “beyond income, beyond averages, beyond today” and focus on inequalities within and across communities^9^.

The Kuznets Curve (KC) is one of the most popular economics concepts for understanding inequality^10^. In the 1950s, Edward Kuznets used an inverted U-shaped curve to depict the hypothesis that as the economy (e.g., income per capita) grows, economic inequality (e.g., inequality of income measured by the Gini coefficient) first increases, then decreases^11^. In the 1990s, the concept of the Environmental Kuznets Curve (EKC) was established^12^.

Similar to the KC, the EKC hypothesis contends that as the economy (e.g., income per capita) grows, environmental degradation first increases then decreases^13^. The EKC has become the main arena of Kuznets-related debates in recent decades^10,14^. EKC-like relationships have since been reported for many environmental quality indicators, especially those for air and water quality that directly impact human health^15^. Air pollution examples include CO_2_, SO_2_, and PM2.5^16–19^; water pollution examples include biochemical and chemical oxygen demand, heavy metals, and organic pollution in water^20,21^. However, neither the KC nor the EKC have been used to investigate the more holistic concept of water security (i.e., the availability, access, use, and reliability of acceptable and safe water)^22^. Moreover, recent research calls for further development of these valuable and vibrant concepts in possible directions such as returning to the original intent of the Kuznets Curve in justice, addressing environmental inequalities in addition to environmental pollution, as well as investigating drivers of inequalities beyond economic indicators^10,23,24^.

**Fig. 1:**
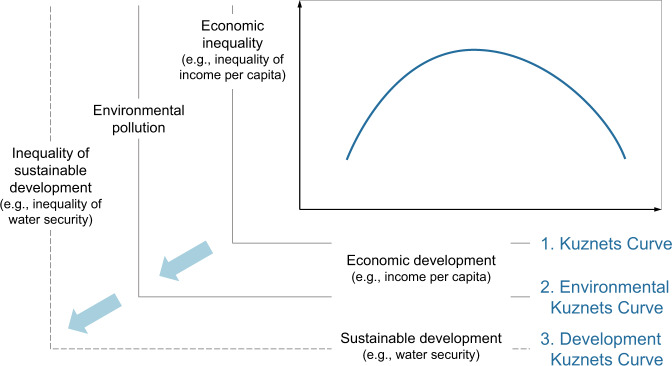
Three stages of Kuznets Curve research. They are Kuznets Curve, Environmental Kuznets Curve, and Development Kuznets Curve.

The KC and EKC are applied in different fields, but both describe an inverted U-shaped relationship, that is, as the independent variable develops, the dependent variable first increases and then decreases (Fig. 1). In both KC and EKC studies, the independent variable describes economic development, which can be measured by income per capita (see x-axis in Fig. 1). The difference between KC and EKC studies is in the dependent variable, which is economic inequality (e.g., income inequality across individuals or Gini coefficient) in KC research^11^ and environmental pollution in EKC applications^25^ (see y-axis in Fig. 1).

Following the above-mentioned possible directions, we set out to test if an inverted U-shaped relationship existed between water security and water security inequality, as an example in the sustainable development context. More specifically, we sought to extend the conventional Kuznets Curve research in three respects, including (1) the choice of indicator, (2) the dependent variable, and (3) the independent variable. These three aspects are expanded on below, and the three-stage development of Kuznets Curve research is illustrated in Fig. 1.

Firstly, regarding the choice of indicators, we use water security indicators to capture environmental and hydrological impacts on human societies. In EKC studies, water is usually understood and assessed in terms of its quality^20,21^ and use^26–28^. According to the DPSIR (driver-pressure-state-impact-response) causal framework^29^, pollution and use are single aspects of environmental pressures or states that do not capture downstream elements such as impacts or responses. Experiential water security more directly and more comprehensively addresses the impacts of water on human health, well-being, and productivity^30–32^, which can also be more relevant to the downstream component responses that tackle the environmental problems^33^. For example, many water security metrics capture accessibility, use, reliability, availability, and safety aspects of water, which have direct impacts on the livelihood at the household and individual levels^22,34^.

Secondly, we expand on existing work by using inequality of water security as the dependent variable to represent inequality of sustainable development, which is developed from economic inequality or environmental pollution in existing Kuznets works (see y-axis in Fig. 1). By doing so, the inequality and environmental elements are combined to address the mounting concerns of water security inequality and justice in the course of sustainable development^35^.

Lastly, we consider water security as an essential facet of sustainable development and use it as the independent variable (see x-axis in Fig. 1). Economic inequality and environmental pollution are assumed to be growth-dependent in the KC/EKC literature. However, economic development may not be the only explanation for the change. Instead, we argue that non-economic factors such as sustainable development levels may also be able to indicate or alter the distribution of resources and services. Therefore, in this study, we test and compare how water security inequality varies by indicators of development, including water security (as a proxy of sustainable development), as well as socioeconomic standing and monthly income (as measures of economic development).

Set against this background, we aim to extend the frontiers of EKC concepts and applications by introducing Kuznets’s thinking to water security and broader sustainable development issues. In principle, the original Kuznets Curve has been explained as a lag-time between the introduction of market-forces and their effects on increasing incomes in a broader population, while the Environmental Kuznets Curve has been explained as a lag-time in technological innovations and regulation changes to cope with environmental degradation. Analogously, we expect to see an inverted U-shaped curve between household water security and its inequality because there is a ‘lag-time’ between the start of local development investment and all households receiving the benefits from the service advancement.

In this work, we show that inequality of household water security follows a Kuznets-like curve, suggesting that as water security grows, the inequality of water security first increases then decreases. Based on this empirical discovery, we extend the original and Environmental Kuznets Curve research and propose the concept of the Development Kuznets Curve to describe the non-linear dynamics of inequality under development beyond economic terms. Finally, we discuss this new concept's theoretical contributions and practical implications in understanding and coping with inequality challenges in the sustainable development context.

## Results

### Relationships between water security and inequality of water security

Most water security indicators assess one or two of the water security domains (e.g., availability, access, quality, reliability, and security) at a macro-level (i.e., the national scale or basin level)^36^. However, such macro-level indicators can mask the heterogeneity and disparities in water availability, access, and reliability experienced by households^34^. Therefore, to obtain higher-resolution data on water (in)security in a cross-culturally equivalent way, the Household Water InSecurity Experience (HWISE) Scale was developed and validated in low- and middle-income countries^37,38^. We used 11 HWISE items to describe household water security (Supplementary Table 1). The Household Water Security (HWS) score is the mean of the 11 items and the Site Water Security (SWS) score is the mean HWS of all households within the site. The inequality of HWS is measured by three indicators including standard deviation (SD), index of ordinal variation (IOV), and polarisation (POL).

We investigated the relationships between water security and inequality of water security by using the HWISE dataset^37^, which consists of household water security and socioeconomic data from 7603 households across 28 sites in 22 low- and middle-income countries throughout Central, South, and Southeast Asia; sub-Saharan Africa; the Middle East; and Latin America and the Caribbean (Supplementary Table 2). We hypothesised that there is (1) an inverted U-shaped relationship between site water security and the inequality of household water security within each site (H1); (2) an inverted U-shaped relationship between site socioeconomic conditions and the inequality of household water security within each site (H2); and (3) a linear relationship between water security and socioeconomic conditions (H3). These hypotheses were tested using 54 regression models (Supplementary Table 3).

**Fig. 2:**
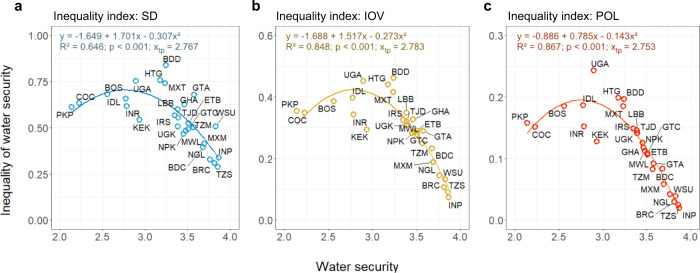
Inverted U-shaped relationship between site water security and the inequality of household water security within each site. The inequality of water security was evaluated by **a** standard deviation (SD), **b** index of ordinal variation (IOV), and **c** polarisation (POL). Sample size = 28. The relationships were tested by quadratic regression models. A more stringent two-sided *p*-value threshold at 0.01 was used to counteract the potential problem of multiple comparisons. The three-figure panels demonstrate similar inverted U-shaped distributions (*p* < 0.001), suggesting a robust and consistent Kuznets-like relationship between water security and its inequality.

The testing of both the relationship between SWS and inequality of HWS and the relationship between each of the 11 water security items and their inequality overall supports H1, suggesting there is an inverted U-shaped relationship between site water security and the inequality of household water security within each site (Fig. 2).

Regarding relationships between SWS and inequality of HWS, Models 1–3 were all significant at *p* < 0.001, and similar quadratic relationships between SWS and the inequality of HWS are captured by the three inequality indicators (Fig. 2; Supplementary Table 4). They predicted turning points at 2.77 (SD), 2.78 (IOV), and 2.75 (POL), which suggests that water security inequality turns from increasing to decreasing when the site reaches a SWS score around 2.8, a relatively high water security level which is at 70% of the upper boundary (score ranges from 0 to 4). Sites UGA (Arua, Uganda) and HTG (Gressier, Haiti) had the highest evaluations of inequality for all three indices, both located around the turning point (SWS of UGA = 2.90; SWS of HTG = 3.18). Site TZS (Singida, Tanzania) had the lowest SD value, and it was among the sites with the highest SWS score. The IOV and POL values of TZS (Singida, Tanzania) are second to INP (Pune, India), which was the most water-secure site. The models using the inequality indicators IOV (R^2^ = 0.848) and POL (R^2^ = 0.867) had higher R-squared values than SD (R^2^ = 0.646) (Fig. 2), suggesting the inequality measures designed for ordinal data generate better regression predictions.

**Fig. 3:**
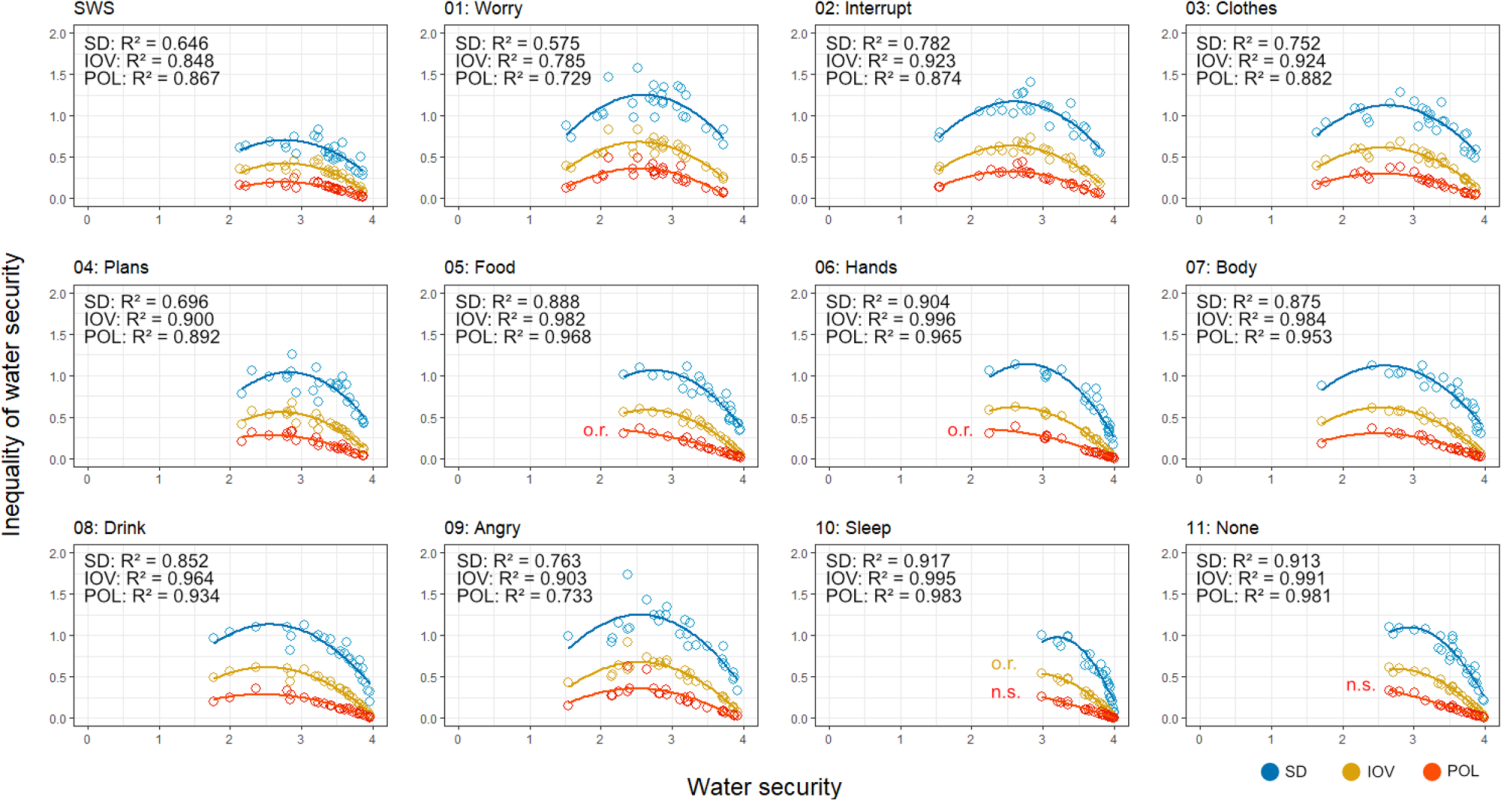
Inverted U-shaped relationship between different water security and inequality indices. Water security indices include composite WS score and the 11 comprising items, and inequality indices include SD, IOV, and POL. Sample size = 28. A more stringent two-sided *p*-value threshold at 0.01 was used to counteract the potential problem of multiple comparisons. n.s. denotes the quadratic regression model is not statistically significant while o.r. denotes the extremum point is out of the range of the available data. See Supplementary Table 4 for details.

Regarding relationships between water security items and inequality of water security items, all 11 water security items followed an inverted U-shaped relationship when SD and IOV were used to measure inequality (*p* < 0.001; Fig. 3; Supplementary Table 4). All but two items (Sleep and None) followed an inverted U-shaped relationship when POL was used as the inequality index (*p* < 0.001). Among the significant inverted U-shaped relationships, the predicted turning points were out of the range of the available data in three items, which were Food (inequality measured by POL), Hands (inequality measured by POL), and Sleep (inequality measured by IOV). The fitted models using IOV and POL generally had a higher R-squared value than the ones using SD, which further suggest that IOV and POL better capture the inequality of water security and the inverted U-shaped relationships than SD does. SD, however, has the highest variation of inequality values among the three indicators, resulting in a higher degree of curvature that is more sensitive to inequality value changes.

### Relationships between inequality of water security and socioeconomic variables

No inverted U-shaped relationships were found between household water security inequality scores (i.e., SD, IOV, and POL) and socioeconomic variables (i.e., SES and INC) (Models 37–42), such that H2 was not supported. Testing of the additional models, however, suggested a significant (*p* < 0.05) linear relationship between water security inequality and SES when inequality was measured by SD (*p* = 0.034, Model 43), IOV (*p* = 0.002, Model 45), and POL (*p* = 0.002, Model 47). These models indicated that sites with higher socioeconomic status have a more equal distribution of water security (*β*_*1*_ *<* *0*). In contrast, no linear relationship was detected between water security inequality and income (Models 44, 46, and 48). Detailed regression results are provided in Supplementary Table 5.

### Relationships between water security and socioeconomic conditions

**Fig. 4:**
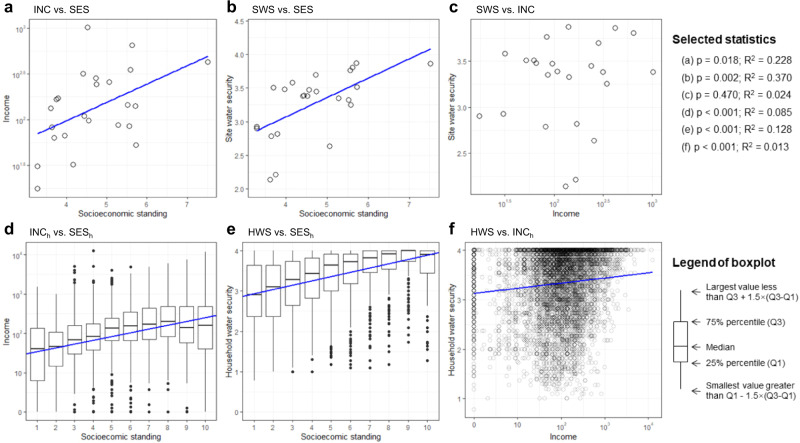
Relationships between income, socioeconomic standing, and water security at the site and household levels. The upper row shows the site-level relationship (*n* = 24). INC, SES, and SWS denote income, socioeconomic standing, and water security at the site level respectively. The bottom row shows the household-level relationship (*n* = 5955). INC_h_, SES_h_, and HWS denote income, socioeconomic standing, and water security at the household level respectively. The relationships were tested by linear regression models and a two-sided *p*-value threshold at 0.05 was used. **a** Income vs. socioeconomic standing at the site level (Model 49). **b** Water security vs. socioeconomic status at the site level (Model 50). **c** Water security vs income at the site level (Model 51). **d** Income vs. socioeconomic standing at the household level (Model 52). **e** Water security vs. socioeconomic status at the household level (Model 53). **f** Water security vs income at the household level (Model 54).

The results of the regression models (Fig. 4; see Supplementary Table 6 for details) were consistent with H3, demonstrating a positive relationship between water security and socioeconomic conditions. Socioeconomic standing and income were correlated at both the site (*p* = 0.018; see Fig. 4a) and household (*p* < 0.001; see Fig. 4d) levels, but they performed differently in the linear models with water security. SES had a linear relationship with both SWS at the site level (*p* = 0.002; see Fig. 4b) and HWS (*p* < 0.001; see Fig. 4e) at the household level. The relationship between SWS and INC, however, was not significant (*p* = 0.470; see Fig. 4c) according to the linear regression model at the site level. Although there was an observed linear relationship between HWS and INC_h_ at the household level (*p* < 0.001; see Fig. 4f), it had a low R-squared value (0.013).

## Discussion

In this suite of analyses, we have responded to recent calls to bring back the original intent of the Kuznets Curve e.g.^10,23,24 ^to expose inequalities and injustices (in this study, by considering disparities in water security at the household resolution). By building quadratic regression models between SWS and inequality of HWS (Model 1–3), and between each water security item and their inequalities (Model 4–36), we discovered a consistent Kuznets-like relationship between site water security and inequality of household water security (Fig. 3, and see full coefficient estimates in Supplementary Table 4). A Kuznets-like curve was found in almost all models, with only three exceptions. All sites had relatively high water security in terms of Food (>2.25/4), Sleep (>2.99/4), and None (>2.66/4), making these three items (i.e., Food, Sleep, and None) unable to capture long enough gradients to cover the middle and low levels of water security, resulting in non-significant curvilinear relationships and out-of-the-range turning points when IOV and POL were used as inequality indicators.

This study opens numerous opportunities for improving our understanding of water security and sustainable development in at least four dimensions: water security assessments, theoretical advancements, empirical studies, and practical implications.

These findings demonstrate the utility of micro-level water security data to quantify and identify water inequalities. Water security can be unevenly distributed, varying dramatically within states, cities, neighbourhoods, and even households^32,37^. Although mitigating inequalities underpins sustainable development and advancing human rights^8^, water security inequality is often overlooked. Understanding and improving inequality challenges of water security require cross-comparable measurements of water security at local, household, and individual levels, instead of conventional measurements at national or basin levels^39^. Such metrics can capture variations across space and people^36^, enabling identification of who exactly is experiencing problems with water and providing more useful information about water security distribution.

Our results encourage a more general rethink of the meaning of the term development. Prior KC/EKC studies follow an economic-centric discourse by investigating the non-linear change of income inequality or environmental variables under economic development. In these studies, economic growth is regarded as the driver of other social or ecological changes^14,40^. This paper provides an example demonstrating the non-linear dynamics of inequality under development beyond economic terms. In this case, development is represented by water security, which is an essential component of sustainable development^41^. Economic development and sustainable variables such as water security are interdependent but refer to different goals and processes. For example, in this paper, water security inequality has an inverted U-shaped relationship with water security level. However, it has no such relationship with the socioeconomic variables (i.e., socioeconomic standing or average monthly income) (Supplementary Table 5), although water security and socioeconomic standing are correlated (Fig. 4).

Therefore, this study proposes a generalised concept of a Development Kuznets Curve (DKC) to describe the change of development inequality in the process of achieving sustainable development goals. This concept goes beyond income inequality, addresses the various experiences and benefits of sustainable development across populations, and considers sustainable development, in addition to economic development, as the underlying factor that brings about transformative changes in resources and services distribution. Sustainable development is a more comprehensive understanding of development than economic growth. Economic growth is regarded as only one of the three pillars of sustainable development, and the other two address environmental (e.g., environmental protection) and social (e.g., social inclusion and justice) dimensions^42^. These calls to end poverty, protect the planet, and ensure people’s well-being are reflected in the 17 UN SDGs^43^. Under this DKC concept, water security is just one case of many possible indicators of resource and well-being that follow the Development Kuznets Curve, making this study a starting point for a more comprehensive examination of Kuznets-like patterns of environmental justice and inequality. Future research may include other aspects of sustainable development, such as inequality in health, education, and hunger^44^. This discovery calls for follow-up studies to further research this hypothetical relationship between sustainable development and inequality/resource measures in more cases (e.g., in terms of household water security and beyond; and sites with different income levels), using observations over time, and more importantly, to reveal the causal mechanisms underlying the new pattern, as for KC and EKC. For example, the KC may be driven by industralialisation, accompanied with the processes of urbanisation, democratisation, and development of welfare systems;^45^ and the EKC can be explained by factors such as income elasticity of demand for environmental quality, scale, technological and composition effects, international trade, and regulations^14,15^. For the household water security case of the DKC, one possible interpretation of the inverted U-shaped relationship is that the improvement of water security (e.g., improved water supply driven by infrastructural development, technologies, or investments) starts from certain households and takes time to reach all households across the whole site. However, additional research is required to test and explain the driving factors of these dynamics as well as the change of inequality beyond economic development^10,35^.

**Fig. 5:**
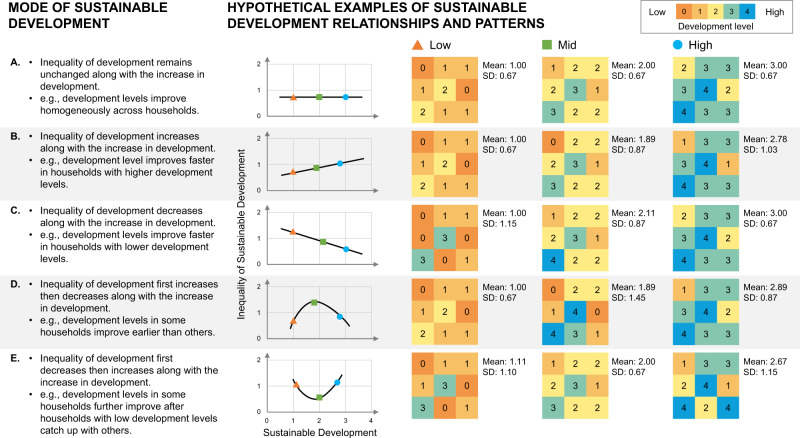
Five modes of sustainable development. For each mode, example patterns of inequality at low, medium, and high levels of sustainable development are illustrated. Household water security is one aspect of sustainable development.

Moreover, the discovered relationship will open a door to improve our understanding of different modes of sustainable development and dynamics of inequalities, including and beyond the inverted U-shaped curve. We, therefore, summarise five basic modes of development, reflecting different hypothetical relationships between sustainable development and inequality of sustainable development (Fig. 5). In the first mode (A), inequality remains unchanged along with development, and development levels improve homogeneously across households. In the second mode (B), inequality increases along with development, and development levels improve faster in households with higher development levels. In the third mode (C), inequality decreases along with development. In this mode, development levels improve faster in households with lower levels of development, and eventually, all households reach a similar high level of development. In the fourth mode (D), inequality first increases then decreases along with development. This mode shows a Kuznets-like relationship between development and inequality of development as we discover and explain using the household water security example in this paper. In this mode, as a combination of Modes B and C, all households have relatively low development levels at the beginning, but the development levels in some households improve earlier than others, and eventually, all households reach a similarly high level of development. In the last mode (E), inequality first decreases then increases along with development. It is a U-shaped relationship, showing development levels in some households further improve after households with low development levels catch up with others. This mode also has two stages and can be seen as a combination of Modes C and B. These five modes are proposed as general hypotheses for future research, especially when cases other than household water security are used.

Some EKC studies demonstrate that the inverted U-shaped relationship may not be accurate if longer-term changes are monitored. Instead, an N-shaped relationship will be detected, indicating the decreased environmental degradation may rise again if the economy continues to grow^15,40^. Recent studies also report that household water security may vary within high-income countries such as the United States^46,47^. This pattern suggests future household water security DKC research should also include study sites from high-income countries in addition to the low- and middle-income ones as used in this paper. If an N-shaped DKC is found, it can be seen as a combination of Modes D and E (see Fig. 5), implying a two-stage development. The inequality of water security first increases then decreases as the benefits of the first stage development gradually reach most households. The inequality of water security increases again when the second stage of development arrives and improves the water security from a proportion of households. Further research is needed to test this alternative DKC pattern.

The findings of this study have important policy implications for managing and improving water security as well as environmental and social sustainability. The UN SDGs call for profound transformation in all countries, and the world has been undertaking economic, political, technological, and social changes to achieve sustainable development^48^. In this development process, resources, services, and rights, as such water security, may improve, change, or redistribute. It potentially contributes to the growing inequality challenge^49^, which is seen as a time bomb of future social unrest^9,50,51^. It is critical that the needs of marginalised people and vulnerable communities for basic services such as water security are heard in policymaking processes to make the community more sustainable^8^. However, the inverted U-shaped relationship discussed herein indicates a potential risk of inflated inequality in the course of sustainable development. This high level of inequality can cause myriad problems. For example, given that psychosocial distress can be caused by perceived injustice, it is likely that communities in the middle of the extremes (i.e., those with the greatest inequality in water security) may have greater levels of distress^52^ or can even precipitate violence^53^, which should be considered and appropriately addressed. Moreover, although the inverted U-shaped relationship globally captures a temporal phenomenon, this newly discovered phenomenon is not necessarily inevitable locally. For example, among the above five modes of development, we should avoid the second mode, in which the improvement of household water security happens in one area but does not further expand across the whole site, making the inequality of water security stay high.

Importantly, the Development Kuznets Curve does not suggest that an increase in inequality is inevitable. Instead, we urge policymakers and funding agencies to fully consider inequality challenges on the way to realising sustainability, such as ensuring water interventions do not further exacerbate entrenched inequalities and avoiding disparities in access and use when expanding water supply networks. We also suggest that more attention is needed among disadvantaged groups, who can be less represented in the governance of water security, especially during the COVID pandemic in which water access was interrupted^6,54^. Likewise, the finding does not imply the inequality problems would disappear effortlessly as a society continues to improve water security^55^. Instead, the Development Kuznets Curve offers a new angle for us to better design sustainable development practices and pathways, including proactive and transformative strategies for achieving fair, balanced, and inclusive development^56–58^.

## Methods

### Household water security

The HWISE Scale queries 12 different experiences with water access and use over the prior four weeks (Supplementary Table 1). An earlier version of the tool that contained only 11 of the 12 final items was administered in 15 out of the total 28 study sites. In regression analyses for sites that implemented the full HWISE Scale, the 11 items asked across all sites accounted for 99.3% of the variation in scales generated using the full 12-item survey (*p* < 0.001). Therefore, we used the 11-item HWISE indicator as a proxy for the validated 12-item HWISE Scale to leverage data across all 28 sites see ^52,59^. Responses to each HWISE item are never (0 days in the last four weeks), rarely (1–2 days), sometimes (3–10 days), often (more than 11 days), or always (more than 20 days). For this analysis, never was scored as 4, rarely as 3, sometimes as 2, often as 1, always as 0, ranging from 0 to 4.

The mean of the 11 items was used to represent the water security of each household (Household Water Security, HWS). For a limited number of households that had missing items, the mean of all surveyed items was calculated as HWS. The Site Water Security (SWS) was calculated by taking the mean HWS of all households within the site. The mean scores of the 11 water security items (i.e., Worry, Interrupt, Clothes, etc.) were also calculated by taking the mean of each item of all households within the site. The SWS score and the 11 water security items are scored at the same scale, ranging from 0 to 4. For both, a larger value indicates a higher water security level. The sample size, mean, and standard deviation of SWS and the 11 items in all study sites are provided in Supplementary Table 2.

### Data collection

Data are from the HWISE study^37^. Approximately 250 households in each of 28 sites across 22 countries in Central, South, and Southeast Asia, sub-Saharan Africa, the Middle East, Latin America, and the Caribbean (Supplementary Table 2; *n* = 7603) were surveyed by trained enumerators in 2017–2018 on household demographics and experiences of water (in)security^38,60^. Sites were selected to maximize heterogeneity of region, urbanicity, water infrastructure, and problems with water. In most sites, households were selected using simple random sampling. However, the data were not collected to be nationally or regionally representative – site labels are at a macro-scale to ensure recognition by most readers without jeopardising participant anonymity. Adults were considered eligible if they reported being knowledgeable about their household’s water situation^37^. Enumerators sought verbal or written informed consent in the respective local language. Study activities were reviewed by all relevant ethical review boards^38^.

### Inequality indicators for water security

Inequalities of income and water security are regarded as the dependent variables in Kuznets Curve research illustrated by Fig. 1. The Gini coefficient is a common indicator for measuring the dispersion of wealth within a group in Kuznets Curve studies^61^. However, unlike income or GDP, data such as self-reported well-being or water security levels are collected in a Likert-style ordinal scale and require alternative approaches for inequality calculation^62^. In this study, we used three inequality measures that are all suitable for ordinal data: standard deviation (SD), index of ordinal variation (IOV), and polarisation (POL). For these indicators, larger values indicate higher inequality.

SD is the most straightforward strategy to measure the inequality of subjective well-being scores as a descriptive statistic and it has been commonly used in studies such as inequality of happiness^62,63^. IOV and POL have been developed for ordinal data, which were meant to address the criticisms on the SD approach for its oversimplified cardinal interpretation as it directly converts the numerical identifier into a cardinal variable^62^. IOV measures the dispersion based on squared Euclidean distances^64^. The POL measures the level of disagreement across respondents^65^. SD, IOV, and POL of household water security within each site were calculated as indicators of water security inequality and are denoted as HWS_SD_, HWS_IOV_, and HWS_POL_.

### Socioeconomic variables

Socioeconomic variables were collected as part of the HWISE study. Perceived socioeconomic standing was assessed using the MacArthur Scale of Subjective Social Status, which asked participants to select which ‘rung on a ladder’ represented where they believed their household stood compared to others in their community (best scored as 10 and worst as 1). Households also reported their average monthly income (converted to US dollars using conversion rates at the end of the sampling period for each site). The income was logarithmised to base 10 to transform to a more normal distribution.

Both subjective social status and reported household income were used to give a fuller picture of socio-economic status. Although the social ladder approach does not capture a household’s objective income, it does, however, provide useful information about how individuals conceptualise their relative standing within their communities and has been shown to be associated with numerous health outcomes (e.g., status syndrome)^66,67^, beyond those predicted by objective socioeconomic status^68–71^. For instance, a household may have high income but be unable to access important resources because of their social standing within the community. However, as in other surveys using this instrument, the term community was not defined and conceptualisations of community may have varied across individuals in the same site, which can be a potential shared limitation of this social ladder approach.

The socioeconomic standing and income data were collected in 5955 households at 24 of the 28 sites (excluding sites BDC, BDD, HTG, and WSU). The household socioeconomic standing score and logarithmised household income are denoted as SES_h_ and INC_h_, respectively (*n* = 5955). The mean of SES_h_ and the mean of INC_h_ within each site are denoted as SES and INC, respectively (*n* = 24).

### Models and hypotheses

A simple reduced-form model was applied to test the relationship between water security and its inequality (see below). This model has been used for most EKC studies^14,15^.

1 $$y={\beta }_{0}+{\beta }_{1}x+\,{\beta }_{2}{x}^{2}+\epsilon$$

where *y* is the inequality of water security within a site (measured by SD, IOV, or POL), *x* is the water security level of the site (i.e., the composite SWS and the 11 comprising items), *β*_0_ is the constant, *β*_1_ is the coefficient of the explanatory variable *x*, *β*_2_ is the coefficient of ${x}^{2}$ (which allows us to examine whether there is a curvilinear relationship between *x* and *y*), and *ϵ* is the random error. Based on this equation, potential relationships include:

1. *β*_1_ = *β*_2_ = 0. No relationship between *x* and *y*.
2. *β*_1_ > 0 and *β*_2_ = 0. A monotonically increasing relationship between *x* and *y*.
3. *β*_1_ < 0 and *β*_2_ = 0. A monotonically decreasing relationship between *x* and *y*.
4. *β*_2_ > 0. A U-shaped relationship between *x* and *y*.
5. *β*_2_ < 0. An inverted U-shaped relationship between *x* and *y*.

Even if (5) is detected, it is still possible that the true relationship is monotonic but not convex if the turning point is predicted to be out of the data availability range^72^. Therefore, we also review the turning point after the detection of (5). The location of the turning point (${x}_{{tp}}$) can be obtained using the equation below.

2 $${x}_{{tp}}=\,-\frac{{\beta }_{1}}{2{\beta }_{2}}$$

The data analysis in this paper was mainly conducted in R 4.0.3^73^, with support of Microsoft Excel (Microsoft 365) and Stata (v16.0). The inequality measures IOV and POL were calculated using the R Package Agrmt 1.42.4^74^.

Three hypotheses (H) were developed to investigate the relationships between water security, inequality of water security, and socioeconomic conditions. They were tested by performing 54 regression models (36 for H1, 12 for H2, and 6 for H3; Supplementary Table 3). The coefficients were considered significant if the two-sided p values were less than 0.05 for H2 and H3. A more stringent *p*-value threshold at 0.01 was used for H1 to counteract the potential problem of multiple comparisons. The coefficient of determination (R^2^) was calculated to evaluate model fit.

**H1:** There is an inverted U-shaped relationship between site water security and the inequality of household water security within each site.

The quadratic relationship was tested using the SWS and the inequality of HWS evaluated by SD, IOV, and POL (Models 1–3). As a sensitivity analysis, we also tested the relationship between each of the mean site water security items (i.e., Worry, Interrupt, etc.) and their inequality within the site. The combination of 11 items and three inequality indices generated 33 models (Models 4–36).

**H2:** There is an inverted U-shaped relationship between site socioeconomic conditions and the inequality of household water security within each site.

H2 was raised to test the existence of a similar quadratic relationship between socioeconomic variables (i.e., SES and INC) and household water security inequality measured by three indices (i.e., SD, IOV, and POL) at the site level (Models 37–42). Additional linear models (Models 43–48) were prepared to further test the relationship between socioeconomic condition and the water security inequality if no quadratic relationship was found in Models 37–42.

**H3:** Water security is positively associated with better socioeconomic conditions.

We hypothesised that water security and socioeconomic conditions (i.e., SES/SES_h_ and INC/INC_h_) would be positively correlated. Two sets of models were constructed to test the relationship at the site level (Models 49–51; *n* = 24 sites) and the household level (Models 52–54; *n* = 5955 households).

### Reporting summary

Further information on research design is available in the Nature Research Reporting Summary linked to this article.

## Supplementary information

Supplementary Information

Reporting Summary

## Data Availability

The sample size, mean, and standard deviation of site water security and the 11 items at each site are provided in Supplementary Table 2. The raw HWISE data are not available in a public data repository due to data sharing restrictions that prevent consent among all HWISE-RCN consortium collaborators. However, the data analysed in this study will be made available upon reasonable request from the HWISE Research Coordination Network (https://hwise-rcn.org/contact-us/).
