# Supplementary material for: Inequality of household water security follows a Development Kuznets Curve
Source: Nat Commun. 2022 Aug 8;13:4525. doi: 10.1038/s41467-022-31867-3 (PMC9360438; doi:10.1038/s41467-022-31867-3)
Supplement: Supplementary file 1 — Supplementary Information [file 41467_2022_31867_MOESM1_ESM.pdf]

Supplementary information

Title

Inequality of household water security follows a Development Kuznets Curve

Authors

Feng Mao, Joshua D. Miller, Sera L. Young, Stefan Krause, David M. Hannah, HWISE Research Coordination Network

This file includes:

Supplementary Tables 1 to 6

Supplementary Table 1. The 12 HWISE Scale items. For each item, questions were asked to elicit the respondents' water (in)security experience in the last four weeks [1]. The first 11 items were used in this study.

No	***Item name***	***Probing question***
1	Worry	How frequently did you or anyone in your household worry you would not have enough water for all of your household needs?
2	Interrupt	How frequently has your main water source been interrupted or limited?
3	Clothes	How frequently have problems with water meant that clothes could not be washed?
4	Plans	How frequently have you or anyone in your household had to change schedules or plans due to problems with your water situation?
5	Food	How frequently have you or anyone in your household had to change what was being eaten because there were problems with water?
6	Hands	How frequently have you or anyone in your household had to go without washing hands after dirty activities?
7	Body	How frequently have you or anyone in your household had to go without washing their body because of problems with water?
8	Drink	How frequently has there not been as much water to drink as you would like for you or anyone in your household?
9	Angry	How frequently did you or anyone in your household feel angry about your water situation?
10	Sleep	How frequently have you or anyone in your household gone to sleep thirsty because there wasn't any water to drink?
11	None	How frequently has there been no useable or drinkable water whatsoever in your household?
12	Shame	How frequently have problems with water caused you or anyone in your household to feel ashamed/excluded/stigmatised?

Supplementary Table 2. The sample size, mean, and standard deviation (SD) of site water security (SWS) and the 11 items. The code is a combination of the two-letter country code and the first letter of the site name.

No	***Code***	***Country***	***Site***	***SWS***			***01: Worry***			***02: Interrupt***			***03: Clothes***			***04: Plans***			***05: Food***		
				n	***Mean***	***SD***	***n***	***Mean***	***SD***	***n***	***Mean***	***SD***	***n***	***Mean***	***SD***	***n***	***Mean***	***SD***	***n***	***Mean***	***SD***
1	BDC	Bangladesh	Chakaria	253	3.68	0.40	253	3.13	1.33	253	3.77	0.57	253	3.87	0.59	253	3.58	0.98	253	3.93	0.43
2	BDD	Bangladesh	Dhaka	253	3.24	0.84	253	2.74	1.24	252	2.71	1.27	251	3.01	1.17	253	3.21	1.09	253	3.21	1.11
3	BOS	Bolivia	San Borja	247	2.56	0.68	238	2.04	1.02	235	2.28	1.05	234	2.26	1.09	227	2.81	0.98	234	2.74	1.00
4	BRC	Brazil	Ceará	254	3.81	0.31	247	3.20	1.24	245	3.58	0.87	246	3.72	0.77	210	3.86	0.47	247	3.96	0.35
5	COC	Colombia	Cartagena	266	2.23	0.63	261	1.51	0.88	242	1.55	0.80	264	1.77	0.92	265	2.31	1.06	255	2.55	1.10
6	ETB	Ethiopia	Bahir Dar	259	3.51	0.52	259	2.87	1.15	10	3.60	0.84	259	3.21	1.00	253	3.47	0.85	259	3.57	0.73
7	GHA	Ghana	Accra	229	3.46	0.62	228	2.88	1.34	222	3.42	0.89	228	3.40	0.93	227	3.56	0.84	227	3.67	0.68
8	GTA	Guatemala	Acatenango	101	3.58	0.68	97	3.08	1.35	100	3.39	1.14	100	3.39	1.16	100	3.63	0.88	100	3.76	0.78
9	GTC	Guatemala	Chiquimula	314	3.51	0.49	313	2.69	1.22	290	2.77	1.06	311	3.12	1.09	312	3.50	0.90	314	3.74	0.63
10	HTG	Haiti	Gressier	292	3.18	0.76	292	2.86	1.26	290	3.01	1.13	292	2.82	1.28	292	2.88	1.25	292	3.39	0.99
11	IDL	Indonesia	Labuan Bajo	279	2.77	0.66	279	1.95	1.00	279	2.10	1.06	277	2.18	1.09	273	2.80	1.01	273	2.94	0.99
12	INP	India	Pune	180	3.87	0.34	179	3.73	0.65	179	3.57	0.75	177	3.80	0.53	179	3.88	0.44	178	3.92	0.42
13	INR	India	Rajasthan	248	2.78	0.62	248	2.46	0.98	238	2.44	1.06	244	2.34	0.97	240	2.55	0.98	243	3.40	0.92
14	IRS	Iran	Sistan & Balochistan	306	3.35	0.57	306	2.76	0.97	136	2.71	1.12	303	3.06	0.87	306	3.24	0.68	303	3.28	0.78
15	KEK	Kenya	Kisumu	247	2.93	0.54	247	2.02	1.04	246	2.59	1.03	247	2.38	0.92	247	2.94	0.79	247	3.15	0.83
16	LBB	Lebanon	Beirut	574	3.39	0.60	573	2.87	1.18	571	3.05	1.11	566	3.21	0.96	570	3.41	0.90	571	3.51	0.85
17	MWL	Malawi	Lilongwe	302	3.48	0.48	301	3.07	1.11	299	2.83	1.07	300	3.23	1.02	301	3.25	0.91	302	3.83	0.56
18	MXM	Mexico	Mérida	250	3.69	0.41	249	2.75	1.37	248	3.32	0.99	247	3.72	0.62	250	3.76	0.64	247	3.94	0.33
19	MXT	Mexico	Torreón	249	3.23	0.74	248	2.75	1.17	247	2.64	1.25	247	3.17	1.06	249	3.51	0.87	249	3.52	0.79
20	NGL	Nigeria	Lagos	239	3.76	0.33	238	3.48	0.84	237	3.43	0.76	238	3.72	0.57	239	3.76	0.53	237	3.81	0.49
21	NPK	Nepal	Kathmandu	263	3.45	0.47	263	2.11	1.47	250	2.83	1.41	262	3.43	0.93	262	3.53	0.87	263	3.81	0.49
22	PKP	Pakistan	Punjab	235	2.14	0.61	235	1.58	0.73	55	1.55	0.74	235	1.63	0.79	233	2.16	0.78	231	2.32	1.01
23	TJD	Tajikistan	Dushanbe	225	3.39	0.55	225	2.52	1.58	225	2.72	1.28	225	3.23	0.90	225	3.64	0.68	224	3.81	0.46
24	TZM	Tanzania	Morogoro	300	3.57	0.50	300	3.15	0.99	283	3.08	1.10	291	3.56	0.86	289	3.68	0.73	283	3.84	0.57
25	TZS	Tanzania	Singida	564	3.86	0.29	564	3.63	0.77	564	3.73	0.63	564	3.85	0.49	564	3.87	0.43	564	3.85	0.46
26	UGA	Uganda	Arua	250	2.89	0.75	248	2.45	1.15	247	2.38	1.14	245	2.67	1.15	247	2.86	1.05	247	3.17	0.97
27	UGK	Uganda	Kampala	246	3.39	0.51	246	3.20	0.98	243	3.09	0.87	244	3.32	0.82	242	3.17	0.82	243	3.41	0.81
28	WSU	Samoa	Upolu	178	3.83	0.51	176	3.72	0.83	175	3.80	0.56	173	3.75	0.79	174	3.86	0.47	174	3.82	0.63

Supplementary Table 2 continued.

No	Code	Country	Site	06: Hands			07: Body			08: Drink			09: Angry			10: Sleep			11: None		
				n	Mean	SD	n	Mean	SD	n	Mean	SD	n	Mean	SD	n	Mean	SD	n	Mean	SD
1	BDC	Bangladesh	Chakaria	253	4.00	0.00	253	3.83	0.65	253	3.82	0.69	253	2.65	1.43	253	3.88	0.56	253	3.98	0.21
2	BDD	Bangladesh	Dhaka	253	3.26	1.07	251	3.13	1.12	253	3.67	0.71	253	3.25	1.15	253	3.93	0.40	253	3.54	0.96
3	BOS	Bolivia	San Borja	236	3.03	1.04	236	2.82	1.03	224	2.36	1.10	232	2.25	1.15	228	2.99	1.00	227	2.66	1.10
4	BRC	Brazil	Ceará	248	3.94	0.40	250	3.86	0.59	254	3.96	0.32	254	3.85	0.55	253	4.00	0.06	253	3.97	0.23
5	COC	Colombia	Cartagena	264	2.62	1.13	265	2.42	1.12	263	2.00	1.04	264	1.55	0.99	266	3.35	0.98	266	2.80	1.09
6	ETB	Ethiopia	Bahir Dar	259	3.87	0.45	259	3.24	0.97	259	3.71	0.66	258	3.49	0.87	259	3.79	0.55	259	3.86	0.42
7	GHA	Ghana	Accra	228	3.75	0.73	228	3.63	0.79	229	3.68	0.72	229	2.92	1.34	229	3.66	0.77	229	3.54	0.85
8	GTA	Guatemala	Acatenango	99	3.75	0.84	99	3.62	0.91	100	3.63	0.92	100	3.72	0.87	101	3.81	0.64	101	3.54	0.99
9	GTC	Guatemala	Chiquimula	314	3.94	0.28	314	3.82	0.51	314	3.55	0.81	314	3.74	0.62	314	3.90	0.43	314	3.76	0.58
10	HTG	Haiti	Gressier	292	3.61	0.84	288	3.42	0.97	290	3.04	1.12	291	3.18	1.18	291	3.35	0.99	290	3.38	1.00
11	IDL	Indonesia	Labuan Bajo	279	3.04	0.98	278	2.79	1.02	279	3.19	0.97	279	2.41	1.09	279	3.72	0.58	279	3.40	0.82
12	INP	India	Pune	177	3.98	0.24	178	3.94	0.30	179	3.91	0.43	180	3.92	0.33	180	3.99	0.07	177	3.88	0.51
13	INR	India	Rajasthan	243	3.05	1.01	240	2.66	1.03	247	2.80	1.10	245	2.16	0.97	242	3.84	0.53	247	3.00	1.06
14	IRS	Iran	Sistan & Balochistan	305	3.59	0.69	306	3.32	0.85	306	3.53	0.77	306	3.34	0.88	306	3.95	0.25	306	3.74	0.53
15	KEK	Kenya	Kisumu	247	3.67	0.63	247	3.15	0.86	247	2.84	0.83	247	2.37	1.08	246	3.65	0.64	247	3.51	0.69
16	LBB	Lebanon	Beirut	566	3.81	0.59	572	3.64	0.73	571	3.41	0.95	570	2.91	1.25	571	3.83	0.53	573	3.61	0.75
17	MWL	Malawi	Lilongwe	300	3.93	0.36	302	3.53	0.82	301	3.60	0.78	300	3.21	1.01	301	3.92	0.39	302	3.81	0.57
18	MXM	Mexico	Mérida	249	3.93	0.32	249	3.89	0.37	249	3.97	0.20	247	3.71	0.71	249	3.98	0.13	247	3.68	0.68
19	MXT	Mexico	Torreón	249	3.61	0.73	248	3.52	0.84	247	3.24	1.01	248	2.81	1.17	247	3.61	0.76	246	3.16	1.07
20	NGL	Nigeria	Lagos	238	3.93	0.35	237	3.89	0.33	236	3.81	0.53	233	3.72	0.66	236	3.94	0.29	235	3.91	0.33
21	NPK	Nepal	Kathmandu	260	3.93	0.32	262	3.74	0.67	263	3.93	0.34	262	2.82	1.25	263	3.96	0.25	263	3.88	0.42
22	PKP	Pakistan	Punjab	229	2.25	1.06	233	1.71	0.88	231	1.77	0.97	230	2.15	0.92	234	3.26	0.87	235	2.71	1.01
23	TJD	Tajikistan	Dushanbe	222	3.98	0.16	225	3.92	0.39	225	3.32	0.97	224	2.38	1.73	225	3.97	0.20	225	3.83	0.44
24	TZM	Tanzania	Morogoro	272	3.90	0.45	291	3.73	0.65	286	3.79	0.61	295	3.12	1.01	272	3.90	0.45	298	3.62	0.78
25	TZS	Tanzania	Singida	564	3.92	0.35	564	3.91	0.39	563	3.86	0.46	564	3.87	0.47	563	3.97	0.21	563	3.97	0.22
26	UGA	Uganda	Arua	246	3.05	0.99	247	3.08	1.00	248	2.86	0.99	245	2.74	1.25	245	3.21	0.92	249	3.35	0.84
27	UGK	Uganda	Kampala	242	3.71	0.59	243	3.58	0.65	242	3.42	0.82	243	3.22	0.87	245	3.60	0.64	227	3.52	0.75
28	WSU	Samoa	Upolu	174	3.84	0.53	173	3.87	0.47	174	3.86	0.60	174	3.88	0.50	174	3.94	0.37	176	3.86	0.63

Supplementary Table 3. Hypotheses to be tested with corresponding regression models.

Hypo.	***Model No.***	***Model***	***Purpose***
H1	1	$HWS_{SD} = \beta_0 + \beta_1 SWS + \beta_2 SWS^2 + \epsilon$	Testing the quadratic relationships between SWS and the inequality of HWS (evaluated by three indices)
	2	$HWS_{IOV} = \beta_0 + \beta_1 SWS + \beta_2 SWS^2 + \epsilon$	
	3	$HWS_{POL} = \beta_0 + \beta_1 SWS + \beta_2 SWS^2 + \epsilon$	
	4-6	$Inequality\ of\ Worry = \beta_0 + \beta_1 Worry + \beta_2 Worry^2 + \epsilon$	Testing the quadratic relationships between the single 11 water security items and the inequality of the 11 items. The inequality of each water quality item was evaluated by three indices (SD, IOV, and POL) respectively.
	7-9	$Inequality\ of\ Interrupt = \beta_0 + \beta_1 Interrupt + \beta_2 Interrupt^2 + \epsilon$	
	10-12	$Inequality\ of\ Clothes = \beta_0 + \beta_1 Clothes + \beta_2 Clothes^2 + \epsilon$	
	13-15	$Inequality\ of\ Plans = \beta_0 + \beta_1 Plans + \beta_2 Plans^2 + \epsilon$	
	16-18	$Inequality\ of\ Food = \beta_0 + \beta_1 Food + \beta_2 Food^2 + \epsilon$	
	19-21	$Inequality\ of\ Hands = \beta_0 + \beta_1 Hands + \beta_2 Hands^2 + \epsilon$	
	22-24	$Inequality\ of\ Body = \beta_0 + \beta_1 Body + \beta_2 Body^2 + \epsilon$	
	25-27	$Inequality\ of\ Drink = \beta_0 + \beta_1 Drink + \beta_2 Drink^2 + \epsilon$	
	28-30	$Inequality\ of\ Angry = \beta_0 + \beta_1 Angry + \beta_2 Angry^2 + \epsilon$	
	31-33	$Inequality\ of\ Sleep = \beta_0 + \beta_1 Sleep + \beta_2 Sleep^2 + \epsilon$	
	34-36	$Inequality\ of\ None = \beta_0 + \beta_1 None + \beta_2 None^2 + \epsilon$	
H2	37	$HWS_{SD} = \beta_0 + \beta_1 SES + \beta_2 SES^2 + \epsilon$	Testing the quadratic relationships between the inequality of HWS and socioeconomic variables. If no quadratic relationships are shown, Model 43-48 will be conducted.
	38	$HWS_{SD} = \beta_0 + \beta_1 INC + \beta_2 INC^2 + \epsilon$	
	39	$HWS_{IOV} = \beta_0 + \beta_1 SES + \beta_2 SES^2 + \epsilon$	
	40	$HWS_{IOV} = \beta_0 + \beta_1 INC + \beta_2 INC^2 + \epsilon$	
	41	$HWS_{POL} = \beta_0 + \beta_1 SES + \beta_2 SES^2 + \epsilon$	
	42	$HWS_{POL} = \beta_0 + \beta_1 INC + \beta_2 INC^2 + \epsilon$	
	43	$HWS_{SD} = \beta_0 + \beta_1 SES + \epsilon$	Testing the linear relationships between the inequality of WS and socioeconomic variables if no quadratic relationships are shown in Model 37-42.
	44	$HWS_{SD} = \beta_0 + \beta_1 INC + \epsilon$	
	45	$HWS_{IOV} = \beta_0 + \beta_1 SES + \epsilon$	
	46	$HWS_{IOV} = \beta_0 + \beta_1 INC + \epsilon$	
	47	$HWS_{POL} = \beta_0 + \beta_1 SES + \epsilon$	
	48	$HWS_{POL} = \beta_0 + \beta_1 INC + \epsilon$	
H3	49	$INC = \beta_0 + \beta_1 SES + \epsilon$	Testing the linear relationships between water security, socioeconomic standing, and income at the site level.
	50	$SWS = \beta_0 + \beta_1 SES + \epsilon$	
	51	$SWS = \beta_0 + \beta_1 INC + \epsilon$	
	52	$INC_h = \beta_0 + \beta_1 SES_h + \epsilon$	Testing the linear relationships between water security, socioeconomic standing, and income at the household level.
	53	$HWS = \beta_0 + \beta_1 SES_h + \epsilon$	
	54	$HWS = \beta_0 + \beta_1 \lg INC_h + \epsilon$	

Supplementary Table 4. Summary of inverted U-shaped curve tests. Two-sided p values were used.

No.	***Model: $y = \beta_0 + \beta_1 x + \beta_2 x^2 + \epsilon$***		***β_1***			***β_2***			***R^2***	***Turning point***	
	x	***y***	***Est.***	***95% C.I.***	***p***	***Est.***	***95% C.I.***	***p***		***Estimate ($-\beta1/2\beta2$)***	***Within the range of observations?***
1	SWS	WS_{SD}	1.701	[0.808, 2.593]	0.001	-0.307	[-0.452, -0.163]	0.000	0.646	2.767	Yes
2	SWS	WS_{IOV}	1.517	[1.078, 1.956]	0.000	-0.273	[-0.344, -0.201]	0.000	0.848	2.783	Yes
3	SWS	WS_{POL}	0.785	[0.561, 1.009]	0.000	-0.143	[-0.179, -0.106]	0.000	0.867	2.753	Yes
4	Worry	$Worry_{SD}$	2.151	[1.357, 2.945]	0.000	-0.415	[-0.563, -0.266]	0.000	0.575	2.594	Yes
5	Worry	$Worry_{IOV}$	1.573	[1.195, 1.951]	0.000	-0.311	[-0.381, -0.240]	0.000	0.785	2.532	Yes
6	Worry	$Worry_{POL}$	1.115	[0.798, 1.432]	0.000	-0.221	[-0.281, -0.162]	0.000	0.729	2.520	Yes
7	Interrupt	$Interrupt_{SD}$	2.045	[1.522, 2.569]	0.000	-0.394	[-0.488, -0.300]	0.000	0.782	2.594	Yes
8	Interrupt	$Interrupt_{IOV}$	1.461	[1.242, 1.681]	0.000	-0.287	[-0.326, -0.248]	0.000	0.923	2.547	Yes
9	Interrupt	$Interrupt_{POL}$	0.942	[0.750, 1.133]	0.000	-0.186	[-0.221, -0.152]	0.000	0.874	2.528	Yes
10	Clothes	$Clothes_{SD}$	1.911	[1.310, 2.511]	0.000	-0.364	[-0.468, -0.259]	0.000	0.752	2.626	Yes
11	Clothes	$Clothes_{IOV}$	1.423	[1.172, 1.675]	0.000	-0.278	[-0.322, -0.234]	0.000	0.924	2.562	Yes
12	Clothes	$Clothes_{POL}$	0.825	[0.629, 1.021]	0.000	-0.163	[-0.197, -0.129]	0.000	0.882	2.536	Yes
13	Plans	$Plans_{SD}$	2.671	[1.392, 3.951]	0.000	-0.474	[-0.678, -0.269]	0.000	0.696	2.820	Yes
14	Plans	$Plans_{IOV}$	1.746	[1.221, 2.270]	0.000	-0.320	[-0.404, -0.237]	0.000	0.900	2.724	Yes
15	Plans	$Plans_{POL}$	0.775	[0.435, 1.114]	0.000	-0.151	[-0.206, -0.097]	0.000	0.892	2.558	Yes
16	Food	$Food_{SD}$	2.445	[1.427, 3.463]	0.000	-0.447	[-0.603, -0.291]	0.000	0.888	2.737	Yes
17	Food	$Food_{IOV}$	1.661	[1.359, 1.962]	0.000	-0.312	[-0.358, -0.266]	0.000	0.982	2.661	Yes
18	Food	$Food_{POL}$	0.296	[0.064, 0.527]	0.014	-0.080	[-0.115, -0.044]	0.000	0.968	1.857	No
19	Hands	$Hands_{SD}$	3.244	[2.036, 4.452]	0.000	-0.585	[-0.769, -0.402]	0.000	0.904	2.770	Yes
20	Hands	$Hands_{IOV}$	1.623	[1.457, 1.789]	0.000	-0.311	[-0.336, -0.286]	0.000	0.996	2.609	Yes
21	Hands	$Hands_{POL}$	0.406	[0.149, 0.663]	0.003	-0.098	[-0.137, -0.059]	0.000	0.965	2.075	No
22	Body	$Body_{SD}$	1.973	[1.378, 2.568]	0.000	-0.381	[-0.478, -0.284]	0.000	0.875	2.587	Yes
23	Body	$Body_{IOV}$	1.361	[1.208, 1.515]	0.000	-0.269	[-0.294, -0.244]	0.000	0.984	2.534	Yes
24	Body	$Body_{POL}$	0.735	[0.583, 0.887]	0.000	-0.147	[-0.172, -0.122]	0.000	0.953	2.501	Yes
25	Drink	$Drink_{SD}$	1.890	[1.242, 2.538]	0.000	-0.369	[-0.476, -0.262]	0.000	0.852	2.561	Yes
26	Drink	$Drink_{IOV}$	1.307	[1.078, 1.535]	0.000	-0.261	[-0.298, -0.223]	0.000	0.964	2.508	Yes
27	Drink	$Drink_{POL}$	0.623	[0.458, 0.788]	0.000	-0.127	[-0.154, -0.100]	0.000	0.934	2.454	Yes
28	Angry	$Angry_{SD}$	2.126	[1.257, 2.995]	0.000	-0.419	[-0.566, -0.271]	0.000	0.763	2.539	Yes
29	Angry	$Angry_{IOV}$	1.567	[1.185, 1.949]	0.000	-0.309	[-0.374, -0.245]	0.000	0.903	2.533	Yes
30	Angry	$Angry_{POL}$	0.934	[0.488, 1.379]	0.000	-0.187	[-0.262, -0.111]	0.000	0.733	2.499	Yes
31	Sleep	$Sleep_{SD}$	7.868	[4.618, 11.117]	0.000	-1.230	[-1.684, -0.775]	0.000	0.917	3.199	Yes
32	Sleep	$Sleep_{IOV}$	2.414	[1.939, 2.890]	0.000	-0.421	[-0.487, -0.354]	0.000	0.995	2.871	No
33	Sleep	$Sleep_{POL}$	0.016	[-0.357, 0.388]	0.932	-0.038	[-0.090, 0.014]	0.146	0.983	0.205	No
34	None	$None_{SD}$	4.431	[2.887, 5.975]	0.000	-0.755	[-0.985, -0.526]	0.000	0.913	2.933	Yes
35	None	$None_{IOV}$	2.006	[1.683, 2.329]	0.000	-0.367	[-0.415, -0.319]	0.000	0.991	2.734	Yes
36	None	$None_{POL}$	-0.098	[-0.351, 0.154]	0.429	-0.023	[-0.061, 0.014]	0.211	0.981	-2.104	No

Supplementary Table 5. Summary of regressions between the inequality of water security (evaluated by SD, IOV and POL) and the socioeconomic variables (i.e., SES and INC). These models were performed for testing H2. Sample size = 24 sites. Two-sided p values were used.

**No.**	***x***	***y***	***β_1***			***β_2***			***R^2***
			Est.	***95% C.I.***	***p***	***Est.***	***95% C.I.***	***p***	
Model: $y = \beta_0 + \beta_1 x + \beta_2 x^2 + \epsilon$									
37	SES	HWS_{SD}	0.073	[-0.328, 0.474]	0.708	-0.013	[-0.052, 0.026]	0.497	0.207
38	INC	HWS_{SD}	-0.218	[-1.364, 0.928]	0.697	0.038	[-0.229, 0.306]	0.770	0.034
39	SES	HWS_{IOV}	0.005	[-0.255, 0.265]	0.968	-0.006	[-0.032, 0.019]	0.605	0.375
40	INC	HWS_{IOV}	-0.237	[-1.065, 0.591]	0.558	0.044	[-0.150, 0.237]	0.643	0.057
41	SES	HWS_{POL}	-0.034	[-0.181, 0.114]	0.642	0.000	[-0.015, 0.014]	0.998	0.364
42	INC	HWS_{POL}	-0.196	[-0.658, 0.267]	0.388	0.039	[-0.069, 0.147]	0.456	0.071
Model: $y = \beta_0 + \beta_1 x + \epsilon$									
43	SES	HWS_{SD}	-0.059	[-0.113, -0.005]	0.034				0.189
44	INC	HWS_{SD}	-0.056	[-0.197, 0.085]	0.420				0.030
45	SES	HWS_{IOV}	-0.060	[-0.095, -0.025]	0.002				0.367
46	INC	HWS_{IOV}	-0.051	[-0.153, 0.051]	0.309				0.047
47	SES	HWS_{POL}	-0.034	[-0.053, -0.014]	0.002				0.364
48	INC	HWS_{POL}	-0.028	[-0.086, 0.029]	0.318				0.045

Supplementary Table 6. Summary of regressions between water security, socioecomic standing and income at both site (n = 24) and household (n = 5,955) levels. These models were performed for testing H3. SWS denotes site water security and HWS denotes household water security. SES and INC denote socioecomic standing and income at the site level, and their household level measurements are denoted as SES_h and INC_h.

No.	***Model: $y = \beta_0 + \beta_1 x + \epsilon$***		***Data level***	***n***	***β_1***			***R^2***
	x	***y***			***Est.***	***95% C.I.***	***p***	
49	SES	INC	Site	24	0.200	[0.037, 0.363]	0.018	0.228
50	SES	SWS	Site	24	0.287	[0.121, 0.453]	0.002	0.370
51	INC	SWS	Site	24	0.174	[-0.317, 0.665]	0.470	0.024
52	SES_h	INC_h	Household	5955	0.096	[0.088, 0.104]	0.000	0.085
53	SES_h	HWS	Household	5955	0.106	[0.099, 0.113]	0.000	0.128
54	INC_h	HWS	Household	5955	0.104	[0.081, 0.126]	0.000	0.013

Supplementary Reference

1. Young, S. L. *et al.* The Household Water InSecurity Experiences (HWISE) Scale: Development and validation of a household water insecurity measure for low-income and middle-income countries. *BMJ Glob. Heal.* **4**, (2019).
