# Supplementary material for: Inequality of household water security follows a Development Kuznets Curve
Source: Nat Commun. 2022 Aug 8;13:4525. doi: 10.1038/s41467-022-31867-3 (PMC9360438; doi:10.1038/s41467-022-31867-3)
Supplement: Supplementary file 2 — Reporting Summary [file 41467_2022_31867_MOESM2_ESM.pdf]

nature portfolio

Corresponding author(s): Feng Mao
Last updated by author(s): 2022-06-14

Reporting Summary

Nature Portfolio wishes to improve the reproducibility of the work that we publish. This form provides structure for consistency and transparency in reporting. For further information on Nature Portfolio policies, see our Editorial Policies and the Editorial Policy Checklist.

Statistics

For all statistical analyses, confirm that the following items are present in the figure legend, table legend, main text, or Methods section.

n/a	Confirmed	
☐	☒	The exact sample size (n) for each experimental group/condition, given as a discrete number and unit of measurement
☐	☒	A statement on whether measurements were taken from distinct samples or whether the same sample was measured repeatedly
☐	☒	The statistical test(s) used AND whether they are one- or two-sided<br>*Only common tests should be described solely by name; describe more complex techniques in the Methods section.*
☒	☐	A description of all covariates tested
☐	☒	A description of any assumptions or corrections, such as tests of normality and adjustment for multiple comparisons
☐	☒	A full description of the statistical parameters including central tendency (e.g. means) or other basic estimates (e.g. regression coefficient) AND variation (e.g. standard deviation) or associated estimates of uncertainty (e.g. confidence intervals)
☐	☒	For null hypothesis testing, the test statistic (e.g. F, t, r) with confidence intervals, effect sizes, degrees of freedom and P value noted<br>*Give P values as exact values whenever suitable.*
☒	☐	For Bayesian analysis, information on the choice of priors and Markov chain Monte Carlo settings
☒	☐	For hierarchical and complex designs, identification of the appropriate level for tests and full reporting of outcomes
☒	☐	Estimates of effect sizes (e.g. Cohen's d, Pearson's r), indicating how they were calculated

Our web collection on statistics for biologists contains articles on many of the points above.

Software and code

Policy information about availability of computer code

Data collection	Data were collected using both paper and tablet-based collection platforms, including Open Data Kit (ODK v1.4; opendatakit.org), CSPro (v7.0; csprousers.org), and KOBOToolbox (v1.27; Cambridge, Massachusetts, USA; kobotoolbox.org). Responses from paper surveys were entered by enumerators, study coordinators, data managers, and/or site primary investigators into an online data collection platform (Enketo; enketo.org). Microsoft Excel was used when reliable internet access was unavailable.
Data analysis	Data were analysed using R (4.0.3), R Package Agrmt (1.42.4), Excel (Microsoft 365), and Stata (v16.0).

For manuscripts utilizing custom algorithms or software that are central to the research but not yet described in published literature, software must be made available to editors and reviewers. We strongly encourage code deposition in a community repository (e.g. GitHub). See the Nature Portfolio guidelines for submitting code & software for further information.

Data

Policy information about availability of data

All manuscripts must include a data availability statement. This statement should provide the following information, where applicable:

- Accession codes, unique identifiers, or web links for publicly available datasets
- A description of any restrictions on data availability
- For clinical datasets or third party data, please ensure that the statement adheres to our policy

The data analysed here will be made available upon reasonable request from the HWISE Research Coordination Network (https://hwise-rcn.org/contact-us/).

Field-specific reporting

Please select the one below that is the best fit for your research. If you are not sure, read the appropriate sections before making your selection.

☐ Life sciences ☒ Behavioural & social sciences ☐ Ecological, evolutionary & environmental sciences

For a reference copy of the document with all sections, see nature.com/documents/nr-reporting-summary-flat.pdf

Behavioural & social sciences study design

All studies must disclose on these points even when the disclosure is negative.

Study description	Household survey data were collected across 28 sites in 22 countries as part of the Household Water InSecurity Experiences project (hwise.org). Data were qualitative and quantitative (i.e., mixed-methods).
Research sample	Approximately 250 households in each of 28 sites across 22 countries in Central, South, and Southeast Asia, sub-Saharan Africa, the Middle East, and Latin America and the Caribbean. Sites were selected to maximise heterogeneity of region, urbanicity, water infrastructure, and problems with water. However, the data were not collected to be nationally or regionally representative. Adults were considered eligible if they reported being knowledgeable about their household's water situation. Only individuals who were knowledgeable about their household's water situation were included since the primary aim of the study was to develop and validate a cross-culturally equivalent tool for measuring household water insecurity. These are primary data.
Sampling strategy	In most sites, households were selected using simple random sampling. The sample size at each site was about 250 households. This sample size is believed as the minimum needed for obtaining a sample pattern that is stable and approximates the population pattern. A sample size calculation was not performed. The target sample size of 250 individuals per site was selected based on published best scale development practices (doi: 10.3389/fpubh.2018.00149).
Data collection	Both study participants and staff were aware that the purpose of the survey was to inform the development and validation of a cross-culturally applicable water insecurity scale. Data were collected using both paper and tablet-based collection platforms, including Open Data Kit (ODK, opendatakit.org), CSPro (csprousers.org), and KOBOToolbox (Cambridge, Massachusetts, USA; kobotoolbox.org). Data were collected on sociodemographic characteristics; water acquisition, use and storage; household food insecurity (using the Household Food Insecurity Access Scale); and perceived stress (using a modified, four-item perceived stress scale). Each interview lasted approximately 45 minutes and data collection lasted approximately 10–14 days in each survey site. Data were collected by trained study staff. Interviews were conducted in private (i.e., only participants and research staff were present). Researchers were not blinded to the study hypotheses.
Timing	Across sites, data were collected between April 2017 and October 2018.
Data exclusions	Individuals with missing data were excluded (i.e., complete-case analysis was used).
Non-participation	We did not collect information on non-participation. When households declined participation, another household was selected until the desired sample size was achieved.
Randomization	This was an observational study (i.e., no randomization).

Reporting for specific materials, systems and methods

We require information from authors about some types of materials, experimental systems and methods used in many studies. Here, indicate whether each material, system or method listed is relevant to your study. If you are not sure if a list item applies to your research, read the appropriate section before selecting a response.

Materials & experimental systems

n/a	Involved in the study	
☒	☐	Antibodies
☒	☐	Eukaryotic cell lines
☒	☐	Palaeontology and archaeology
☒	☐	Animals and other organisms
☐	☒	Human research participants
☒	☐	Clinical data
☒	☐	Dual use research of concern

Methods

n/a	Involved in the study	
☒	☐	ChIP-seq
☒	☐	Flow cytometry
☒	☐	MRI-based neuroimaging

Human research participants

Policy information about studies involving human research participants

Population characteristics	Respondents were adults who identified as being familiar with their household's water situation. Across sites, the majority of participants were female and the mean age was 39, although there was substantial heterogeneity across settings.
Recruitment	Sites with simple randomized sampling employed a random-walk sampling method. With the simple randomized sampling strategy, a random number generator (e.g., dice or random number generating application) with set parameters (i.e., less than 20, less than 30 and so on) determines which households to survey. For sites using a cluster-randomized sampling [illegible]

	strategy, the region is first mapped using a grid or satellite imagery (e.g., Google Maps) to identify population density based on the number of habitable structures. Clusters are selected from this grid, and households within clusters are randomly sampled in proportion to structure or population density using a random number generator, similar to the simple randomized sampling strategy. Adults were eligible for survey inclusion if they considered themselves to be knowledgeable about water acquisition and use in their household. Participants gave oral or written informed consent.
Ethics oversight	Study activities received necessary ethical approvals from institutional review bodies relevant to each site: Texas A&M University, Northwestern University, Pontificia Universidad Javeriana, Arizona State University, McGill University, University of Florida, Ghana Water Company, University of Lagos, Oregon Health Sciences University, African Medical Research Foundation, Cornell University, T-Group Kampala, Georgia State University, Sokoine University of Agriculture, American University of Beirut, and Yale University.

Note that full information on the approval of the study protocol must also be provided in the manuscript.
